# Differences in plasma proteomes for active tuberculosis, latent tuberculosis and non-tuberculosis mycobacterial lung disease patients with and without ESAT-6/CFP10 stimulation

**DOI:** 10.1186/s12953-020-00165-5

**Published:** 2020-10-31

**Authors:** Takele Teklu, Biniam Wondale, Biruhalem Taye, Milkessa Hailemariam, Shiferaw Bekele, Mesfin Tamirat, Aboma Zewude, Temesgen Mohamed, Girmay Medhin, Mengistu Legesse, Yanbao Yu, Gobena Ameni, Rembert Pieper

**Affiliations:** 1 Department of Immunology and Molecular Biology, University of Gondar, Gondar, Ethiopia; 2 Aklilu Lemma Institute of Pathobiology, Addis Ababa University, Addis Ababa, Ethiopia; 3 Department of Biology, Arba Minch University, Arba Minch, Ethiopia; 4 European Molecular Biology Laboratory, Notkestraβe 85, 22607 Hamburg, Germany; 5 Department of Veterinary Laboratory, Ambo University, Guder, Ethiopia; 6 J. Craig Venter Institute, Rockville, MD USA; 7 Laboratory department, Jinka General Hospital, Jinka, Ethiopia; 8 Ethiopian Public health Institute, P.O box 1242, Addis Ababa, Ethiopia; 9 Department of Veterinary Medicine, College of Food and Agriculture, United Arab Emirates University, P.O. Box 15551, Al Ain, United Arab Emirates

**Keywords:** MTBC, NTM, Blood plasma, ESAT-6/CFP-10 antigen cocktail, Proteomics, Protein biomarker, LC-MS/MS

## Abstract

**Background:**

Tuberculosis (TB) is one of the world’s most problematic infectious diseases. The pathogen *Mycobacterium tuberculosis* (*Mtb*) is contained by the immune system in people with latent TB infection (LTBI). No overt disease symptoms occur. The environmental and internal triggers leading to reactivation of TB are not well understood. Non-tuberculosis Mycobacteria (NTM) can also cause TB-like lung disease. Comparative analysis of blood plasma proteomes from subjects afflicted by these pathologies in an endemic setting may yield new differentiating biomarkers and insights into inflammatory and immunological responses to *Mtb* and NTM.

**Methods:**

Blood samples from 40 human subjects in a pastoral region of Ethiopia were treated with the ESAT-6/CFP-10 antigen cocktail to stimulate anti-*Mtb* and anti-NTM immune responses. In addition to those of active TB, LTBI, and NTM cohorts, samples from matched healthy control (HC) subjects were available. Following the generation of sample pools, proteomes were analyzed via LC-MS/MS. These experiments were also performed without antigen stimulation steps. Statistically significant differences using the Z-score method were determined and interpreted in the context of the proteins’ functions and their contributions to biological pathways.

**Results:**

More than 200 proteins were identified from unstimulated and stimulated plasma samples (UPSs and SPSs, respectively). Thirty-four and 64 proteins were differentially abundant with statistical significance (*P* < 0.05; Benjamini-Hochberg correction with an FDR < 0.05) comparing UPS and SPS proteomic data of four groups, respectively. Bioinformatics analysis of such proteins via the Gene Ontology Resource was indicative of changes in cellular and metabolic processes, responses to stimuli, and biological regulations. The m7GpppN-mRNA hydrolase was increased in abundance in the LTBI group compared to HC subjects. Charged multivesicular body protein 4a and platelet factor-4 were increased in abundance in NTM as compared to HC and decreased in abundance in NTM as compared to active TB. C-reactive protein, α-1-acid glycoprotein 1, sialic acid-binding Ig-like lectin 16, and vitamin K-dependent protein S were also increased (*P* < 0.05; fold changes≥2) in SPSs and UPSs comparing active TB with LTBI and NTM cases. These three proteins, connected in a STRING functional network, contribute to the acute phase response and influence blood coagulation.

**Conclusion:**

Plasma proteomes are different comparing LTBI, TB, NTM and HC cohorts. The changes are augmented following prior blood immune cell stimulation with the ESAT-6/CFP-10 antigen cocktail. The results encourage larger-cohort studies to identify specific biomarkers to diagnose NTM infection, LTBI, and to predict the risk of TB reactivation.

## Background

Tuberculosis (TB) is an ancient disease caused by bacteria of the *Mycobacterium tuberculosis* complex (*Mtb*) and continues to be an urgent public health problem. In 2016 alone, 10.4 million new infections and 1.7 million deaths were reported [1]. The host immune response mechanisms are not well understood despite decades of research, and the improvement of diagnostics, therapeutics, and vaccines remain biomedical priorities worldwide. To develop better diagnostics, molecular biomarkers, and methods to discover them need to be identified [2]. Using blood plasma from active TB, latent TB infection (LTBI), and healthy control (HC) subjects as sample sources, shotgun proteomic comparisons may lead to new biomarkers, yield information on mechanisms underlying differences in disease outcomes and contribute to future strategies to prevent and treat TB.

Non-tuberculosis Mycobacteria (NTM) are environmental microorganisms that belong to the genus *Mycobacterium*. NTM can cause lung disease in humans clinically similar to TB [3] but is often treated differently [4]. Previous exposure to NTM reduces the efficacy of the BCG vaccine [5, 6]. Infection with NTM results in a false negative value using the purified protein derivative (PPD) test. Conventional diagnosis of TB and NTM depends on culturing the bacteria in specific growth media, but culture-based identification methods in conjunction with biochemical tests are slow and do not adequately speciate the pathogens [7]. More rapidly measurable biomarkers that differentiate NTM from *Mtb* infections and corresponding diagnostic assays with high measurement sensitivity and specificity are clinically valuable. Such diagnostic tests may benefit approaches for therapeutic intervention and pathogen transmission control [8]. Laboratory methods to identify Mycobacteria from clinical specimens begin to result in faster and more accurate identification of NTM strains [4]. Research that advances diagnostic capabilities for NTM infections is important because the incidence of NTM infections appears to increase, and the level of protection of BCG-vaccinated individuals against such infections is low [9].

Major challenges of conventional biomarker discovery research are limited insights into disease mechanisms and high dependence on cohort design, sample type, analytical and statistical approaches. In the TB context, for example, Gerhard et al. [10] stated that the assessment of the risk of infection versus protection from TB should include data-driven methods, such as global ‘omics’ screens, in addition to the generation and interpretation of immunological profiles. For example, the measurement of multiple cytokines from individual patients selectively applied to distinct T-cell populations likely is more informative to distinguish LTBI from active TB than the measurement of a single cytokine from pools of T-cells. Confounding factors for successful biomarker discovery are high diversity within cohorts with respect to socio-demographics, genetics, and medical history and the reliance on single as opposed to multiple analyte measurement time points. Prior courses of anti-TB drug treatment and other medications used by patients also influence susceptibility to infection and disease severity. With respect to ‘omics’ screens, it was reported that transcriptional signatures distinguish active TB from LTBI [11, 12] and can predict the progression of LTBI to active TB [13]. Translating such signatures into diagnostic tests can be challenging. mRNAs have high turnover rates. Preserving the analytes prior to measurement can be technically difficult, especially in a resource-poor setting. Since proteins are more stable analytes and can be measured with good quantitative accuracy in a highly parallel manner from clinical sample sources (e.g. body fluids), proteomics is a promising technique to discover biomarkers that distinguish outcomes of disease or predict the risk of disease onset or severity [14, 15]. Blood plasma is a common source of specimens.

Proteomic surveys to identify blood plasma or serum biomarkers for TB and antibiotic treatment outcomes were applied to different cohorts and generated variable results. Unstimulated plasma samples (UPS) have been used to compare proteomic data from active TB and HC cohorts [16, 17]. Blood plasma samples stimulated with TB-specific antigens (SPS) [18] have been used to discern latency from the active disease in a high TB burden country [19]. Antigens commonly used to elicit TB-specific immunological responses in the blood are ESAT-6 and CFP10. They are the basis of Interferon Gamma Release Assays (IGRA) to diagnose LTBI. It was reported that the BCG vaccine strain and most NTM strains do not harbor the ESAT-6 and CFP10 antigens, leading to different immune cell responses to ESAT-6 exposure [20, 21]. Important objectives are to characterize the utility of IGRAs for the differential diagnosis of NTM infections and to assess whether TB latency elicits immune and metabolic changes other than the release of IFN-γ. Chegou et al. used the IGRA assay product QuantiFERON-TB to measure proteins in blood stimulated with TB antigens, but did not conduct the study in comparison to a HC cohort [22]. To our knowledge, our study is the second one to investigate plasma proteomic profiles from patients with pulmonary TB compared to LTBI using UPSs [23] and the first one to use mass spectrometry-based proteomics that also includes SPSs as a sample source. We intended to gain insights into plasma proteome differences using two types of samples (UPSs and SPSs) from four distinct cohorts (HC, active TB, LTBI, and NTM) and identify preliminary protein biomarkers that discern the pathologies.

## Materials and methods

### Study subjects and data collection

Patients with active pulmonary TB were recruited from local clinics caring for TB patients in selected districts in the South Omo Zone. Blood samples were collected from patients with pulmonary TB before treatment initiation. LTBI and healthy controls (HC) were recruited from the same districts and screened using the QuantiFERON-TB Gold In-Tube test (QFT-IT) as described in a previous study [24]. Clinical data were collected to rule out the possibility that the control and LTBI groups had clinical signs and symptoms of other respiratory diseases [25]. The active TB group was identified using a standard smear microscopy test for acid-fast bacilli and the mycobacterial culture method. Genus typing was done using DNA amplification in a single tube, in addition to culture-positive results, to categorize taxa into *Mtb* and NTM [26]. Individuals who had complications attributable to malignancies, autoimmune diseases, or HIV co-infection were excluded from the study.

Blood samples were collected from 40 enrolled participants (10 each for the cohorts HC, LTBI, active TB, and NTM) into vacutainer tubes (Becton Dickinson, Franklin Lakes, NJ, USA) without anticoagulant and allowed to clot at room temperature for 1 h. The clotted samples were centrifuged at 1500×*g* for 10 min to separate the soluble fraction (serum). Sera were immediately aliquoted into sterile tubes and stored at −80^о^C prior to further use. Whole blood samples were collected in parallel and stimulated with ESAT-6/CFP-10 cocktail antigens as previously described [24]. Briefly, 1 mL of heparinized whole blood was diluted in RPMI 1640 medium containing L-glutamine (Sigma) supplemented with penicillin, 100 U/ml, and streptomycin, 100 μg/ml (Sigma) to a final dilution of 1:10. This ESAT-6/CFP-10 cocktail was used to stimulate whole blood at a final concentration of 10 μg/ml. For positive and negative controls, phytohemagglutinin antigen (PHA) at 10 μg/ml (Sigma) and RPMI 1640 media were used, respectively. After 48 h of incubation at 37 °C with 5% CO_2_, supernatants were harvested and stored at − 80 °C until further use. Sample preparation for proteomics pertained to the generation of sample pools (ten samples of equal volumes in a given plasma sample pool). There were eight plasma pools: HC, LTBI, active TB, and NTM group, each with two sample types (UPS and SPS).

### Depletion of high-abundance plasma proteins

Each UPS and SPS sample pool was subjected to immunoaffinity depletion of 11 abundant human plasma proteins using a chromatographic matrix. This matrix consisted of a pool of modified POROS-A resins, each containing covalently immobilized polyclonal antibodies. This matrix was used in a batch mode to bind and elute protein and removed up to 95% of each protein. Depletion efficiency varied based on (1) polyclonal antibody specificity, (2) stability and retention of antibodies on the recycled matrix, and (3) target protein solubility in the cycle of binding at neutral pH and elution at pH 2.2. The affinity targets were: IgG (which binds to the *Staphylococcus aureus* protein A directly bound to the product POROS A), albumin, antitrypsin, IgA, transferrin, haptoglobin, fibrinogen, alpha-2-macroglobulin, IgM, apolipoprotein AI, apolipoproteinAII, complement factor C3, and transthyretinas previously described [27]. The total protein concentration of the flow-through plasma fraction from the resin was determined by the Bradford assay technique and SDS-PAGE gel electrophoresis. Filter aided sample preparation (FASP) [28] using a 30 kDa cutoff filter was used for digestion with minimal modifications [29]. Using approximately 50 μg total plasma protein, tryptic digestion was followed by peptide desalting via the spinnable Stage Tip protocol [30]. Dried peptides were resuspended in 20 μl solvent A (0.1% formic acid in water) for LC-MS/MS analysis.

### LC-MS/MS and proteomic data analysis

LC-MS/MS experiments were performed using an Ultimate 3000-nano LC system coupled to a Q-Exactive mass spectrometer (Thermo Scientific). The experimental and data acquisition methods were previously described in detail [29]. Briefly, peptides were separated over a 150 min gradient from 2 to 80% (120 min to 35%, 10 min to 80%) in solvent B (0.1% formic acid in acetonitrile) at a flow rate of 200 nl/min in an in-house packed column (75 μmx 18 cm, 3.0 μm ReproSil-Pur C18-AQ). The MS survey scans were acquired at a resolution of 70,000 over a mass range of m/z 250–1,800 with an automatic gain control (AGC) target of 1e6. The maximum injection time (IT) was 30 ms. In each cycle, the ten most intense ions were subjected to high-energy collisional dissociation (HCD) applying normalized collision energy of 27%. The MS/MS scans were performed at a resolution of 17,500. The AGC target was set to 2e5 and the maximum IT was 150 ms. Charge exclusion included + 1 and + 5 or more. Dynamic exclusion of repeated MS1 peaks was enabled (exclusion from MS/MS after 20 s). Pooling technical LC-MS/MS replicates, the MS raw data files were processed using the Sequest HT algorithm integrated into the Proteome Discoverer software analysis platform (version 1.4, Thermo Scientific). A database that contained protein sequences from the *Mtb* strain ATCC 25618 / H37Rv (7,955 sequences) and a non-redundant human proteome database subset (20,195 sequences; reviewed sequences only; version 2015_06) obtained from the UniProt knowledgebase was used to computationally identify peptides and proteins. Search parameters included (1) two missed tryptic cleavages, (2) oxidation (M), protein N-terminal acetylation and deamidation (N, Q) as variable modifications, and (3) carbamidomethylation (C) as a fixed modification. The minimum peptide length was seven amino acids. MS and MS/MS ion Proteome Discoverer tolerances were set at 10 ppm and 0.02 Da, respectively. The FDR was estimated using the integrated Percolator tool. Only protein hits identified with a 1% FDR threshold were accepted. For protein quantification, the MaxQuant and Andromeda software suite (version 1.4.2.0) was used. We accepted most of the default settings provided in this software [31]. A 1% FDR was set at both the peptide and protein level. The MaxLFQ algorithm generates relative quantification values using the integrated MS1 peak areas from high-resolution MS data [32]. The clustering and correlation analyses were performed in the Perseus environment (version 1.5.0.15) using embedded functions [33]. Before analysis, the LFQ intensities generated by MaxQuant were log (base 2) transformed, and then imputed with missing values by default settings in Perseus. LC-MS/MS data were deposited to the Proteome Xchange Consortium via the PRIDE partner repository with the dataset identifier PXD012412. Detailed data on protein/peptide identifications are provided in Supplemental Data, Dataset S1.

### Bioinformatics and statistical analysis

Differences in protein abundance among the active TB, LTBI, NTM, and healthy subject cohorts were compared by one-way analysis of variance (ANOVA) using the IBM SPSS software version 20. Multiple comparisons were performed using the least significant difference (LSD) post-hoc test when the variance between samples was equal or the Dunnett’s T3 test and when the variances were not equal. The independent-sample *t*-test was used to identify differences in relative analyte abundance levels comparing active TB with the combination of the other three groups. Comparisons of data pertaining to SPSs (stimulated) and UPSs (unstimulated samples) in the assays were performed using a paired-sample *t*-test. *P-*values < 0.05 were considered statistically significant. The Perseus 1.5.0.15 software was used for graphing heat maps. Cellular component, molecular function, and biological process categories were assigned using the Gene Ontology Resource (http://www.geneontology.org/). Signaling pathways that proteins contribute to were assessed by searching against the Kyoto Encyclopedia of Genes and Genomes database (http://www.genome.jp/kegg/pathway.html). Protein-protein interaction network analyses were derived from the Search Tool for the Retrieval of Interacting Genes/Proteins (STRING) software (http://string.embl.de/).

### Ethics statement

Ethical approval for the study was obtained from Addis Ababa University, Aklilu Lemma Institute of Pathobiology Research and Ethics Committee as well as from the National Research Ethics Committee of Ethiopia (Ref No:3.10/785/07). Written consent was obtained from each study participant after clearly explaining the objective of the study. Blood sample collection was undertaken under aseptic conditions by licensed medical laboratory professionals. Volunteer individuals with signs and symptoms of active TB or any other disease during the enrolment period were treated in nearby health facilities at the expense of this study.

## Results

### Socio-demographic characteristics of study participants

Of the 40 study participants, 5 (50%), 5 (50%), 5(50%) and 5 (50%) were males for HC, LTBI, active TB cases and NTM cases, respectively. There was no significant gender difference in the four groups (χ^2^ = 0.220; *P* = 0.896). The mean age of healthy individuals was 35.71 with an SD of 12.52, the mean age of individuals with LTBI was 37.15 with an SD of 11.69, the mean age of individuals with active TB was 34.52 years with an SD of 17.48; the mean age of individuals with NTM was 34.71 with an SD of 12.52. There was no significant age difference among the four groups (*P* = 0.685). Healthy and LTBI subjects were free of TB clinical signs and symptoms. All individuals with active TB and NTM infections had clinical signs and symptoms of TB. There was a significant mean IFN-γ value difference comparing the four groups (*P* < 0.001) (Table 1).

**Table 1:** Study participant traits

Subject Characteristics	HC	LTBI	Active TB	NTM	P-Value
Male to female ratio	1:1	1:1	1:1	1:1	0.90
Mean age (SD)	35.71 (12.52)	37.15 (11.69)	34.52 (17.48)	34.71 (12.52)	0.69
TB symptoms, yes (%)	–	–	10 (100%)	10 (100%)	–
Mean IFN-*γ* values	347.57	908.38	531.31	710.78	< 0.001

### Identification and relative quantification of proteins in unstimulated plasma

**Fig. 1:**
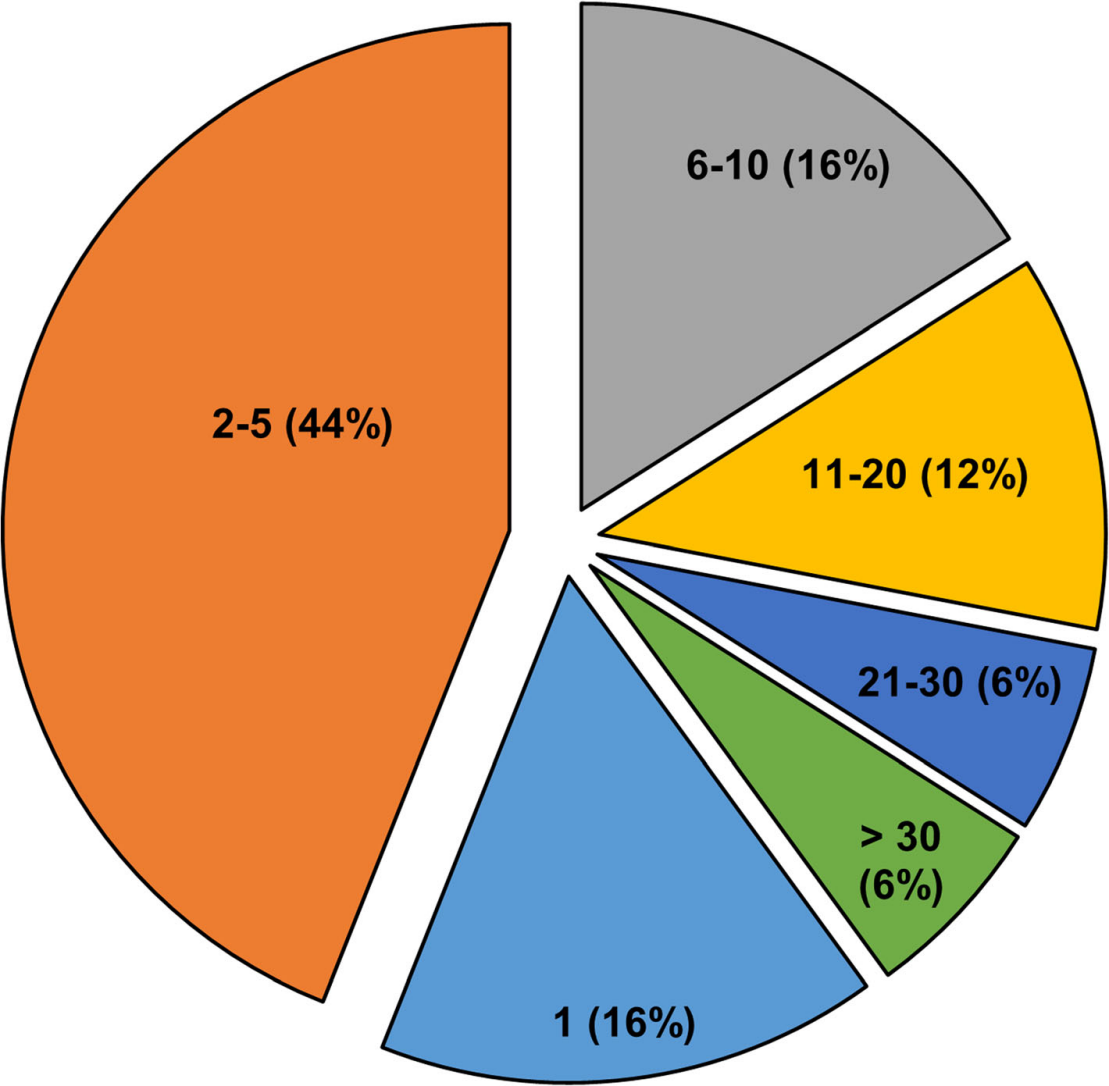
Distribution of the number of unique peptides quantified per protein for the combined unstimulated and stimulated plasma proteomic datasets. To quantify total number of proteins first, all the identifications were examined and assessed the number of peptides per protein. Over 84% of the total (189) quantifiable proteins were quantified based on two or more peptides. For downstream analysis, we further filtered the data to require the proteins to be quantified in at least 3 out of 8 LCMS runs. Among the resulting 160 proteins, 152 (95%) were quantified based on at least 2 unique peptides. Unique proteins reported in the manuscript were unique protein groups

**Fig. 2:**
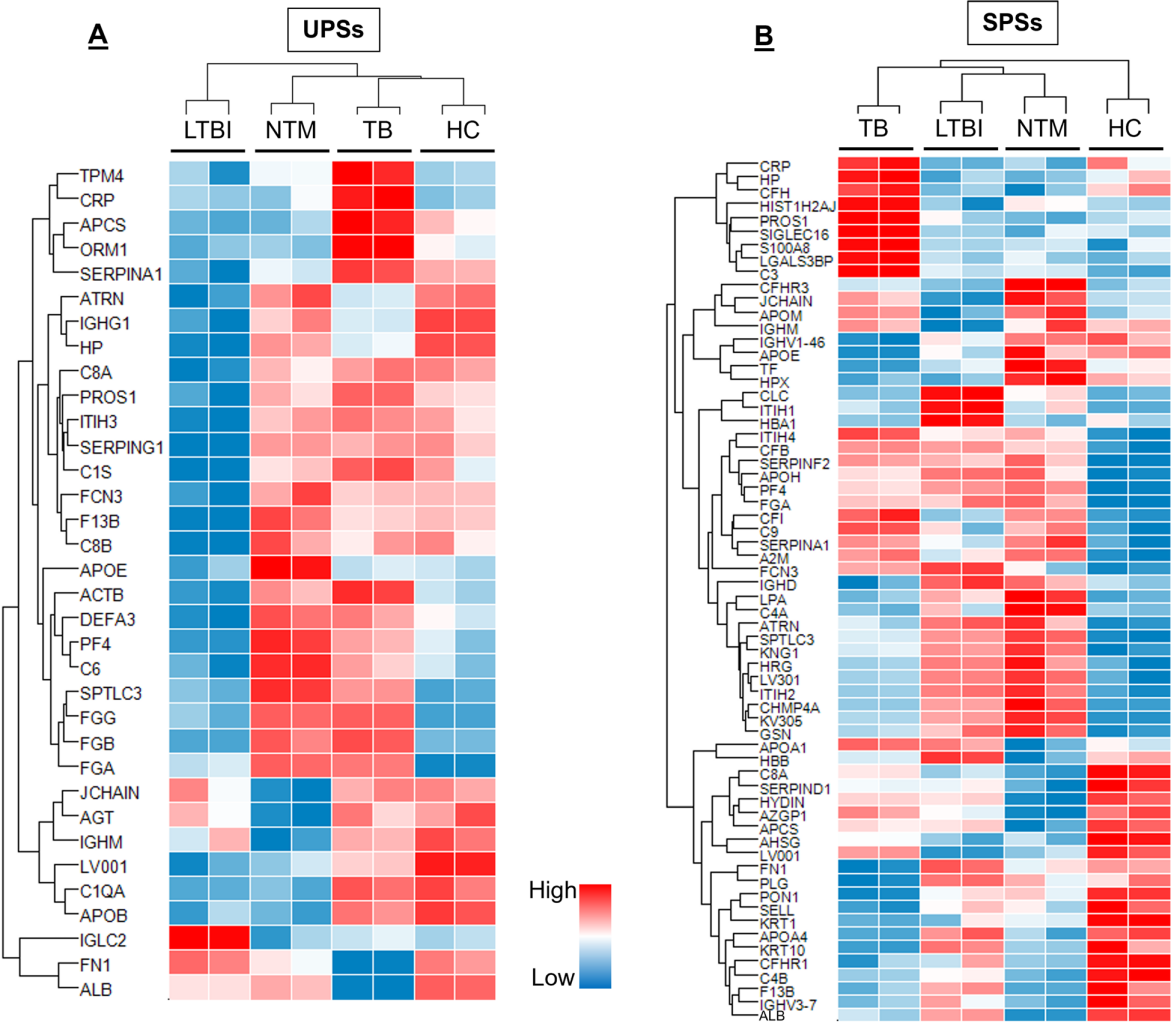
**a** Label-free quantitative proteomic analysis of plasma samples, in pools of ten patients each, from (1) Mtb-caused TB, (2) NTM-caused TB, (3) LTBI, and (4) healthy controls. Proteins are listed with their UniProt short names (UniProt.org). Thirty-four proteins showed significant differences among the four groups. The proteins were plotted in the heatmap after Z-score normalization and unsupervised hierarchical clustering. Both UPS and SPS profiles are displayed with two LC-MS/MS replicates (rep1, rep2) for each of the four groups

The UPS samples were analyzed using a well-established high-resolution accurate mass (HR/AM) based LC-MS/MS system [30, 34, 35] without pre-fractionation in two technical replicates. Using the MaxLFQ algorithm 189 proteins were quantified (Supplemental Table-S2); 152 proteins were present in at least 3 of the 8 analyses and quantified based on at least two unique peptides. The distribution of the number of unique peptides quantified per protein for the UPS data is illustrated in Fig. 1. Following multiple-sample statistical tests (ANOVA), 34 proteins were differentially abundant with correction for multiple testing using the Benjamini-Hochberg method (FDR ≤0.05). The unsupervised hierarchical clustering analysis presented in Fig. 2a includes proteins with statistically significant differences in abundance for all four cohorts under study. Independent-sample *t*-tests were used to identify these proteins (LTBI versus HC, NTM versus HC, and active TB versus HC; Supplemental Table-S2). In all cases *P*-values < 0.05, q-values < 0.05, and fold changes of t-test difference at + 1 or − 1 were considered significant. Four proteins were increased in abundance, and 31 proteins were decreased in abundance in the LTBI group compared to the HC group. M7GpppN-mRNA hydrolase (DCP2) is one of the proteins that increased in the LTBI cohort. Twenty-four proteins were increased in abundance, and 19 proteins were decreased in abundance in the NTM vs. HC cohort. Twenty-nine proteins were increased in abundance (four of which were also identified in NTM cases) and 20 proteins were decreased in abundance in active TB cases compared to the HC group. Independent-sample *t*-tests were also used to identify proteins with statistically significant differences in LTBI vs. NTM and LTBI vs. active TB. Twenty-nine proteins were increased and five proteins were decreased in abundance with statistically significant differences between the LTBI and NTM cohorts. Thirty-seven proteins were increased in abundance and four proteins were decreased in abundance in active TB as compared to LTBI. Sixteen proteins were increased in abundance and 22 proteins were decreased in abundance in active TB as compared to NTM. One cluster of proteins more abundant in active TB compared to the other groups (α-1-acid glycoprotein 1 (ORM1), C-reactive protein (CRP), sialic acid-binding Ig-like lectin16 (SIGLGC16), and serum amyloid A-1 protein (SAA1) is enriched in proteins functionally associated with the acute phase (inflammatory) response. A cluster of four proteins consists of fibrinogen subunits and serine palmitoyltransferase 3 (SPTLC3). Fibrin is responsible for coagulation of blood while SPTLC3 metabolizes sphingolipids which, in turn, alter protease activities involved in coagulation. Those proteins are more abundant in TB and NTM cases compared to the LTBI and HC groups.

### Protein biomarkers in stimulated plasma

SPS proteomic analyses resulted in a total of 190 identified proteins (including proteins with a single unique peptide) 144 proteins were quantified (Fig. 2b). ANOVA analysis revealed 64 proteins significantly different in abundance comparing the groups (active TB, NTM, LTBI, and HC) with a Z-Score *>* 1.5, *P <* 0.05 (FDR; *q* 0.05), applying the Benjamini-Hochberg multiple testing correction. The data suggests group-specific adaptations in the plasma proteome upon ESAT-6/CFP10 stimulation. The proteomes of the four subject groups clustered differently from those observed for UPSs. Not unexpectedly, the HC proteome forms a cluster more distant from those of the other groups in SPSs, as compared to UPSs. Independent-sample *t*-tests were used to compare changes in abundance of proteins comparing two groups (Supplemental Table-S2). In all cases *P*-values < 0.05, q-values < 0.05, and fold changes of t-test difference at + 1 or − 1 were considered significant. Thirty-two proteins were increased and 17 proteins were decreased in abundance in the LTBI compared to the HC cohorts. Among them were the platelet factor (PF)-4, DCP2, SPTLC3, kininogen-1, and ficolin-3. Twenty-five proteins were increased in abundance, and 16 proteins were decreased in abundance in the NTM versus HC groups. For the LTBI and NTM datasets, most proteins that were differentially abundant in comparison with HC datasets matched. Sixty-one proteins were differentially regulated comparing the active TB and HC groups, 33 of which were increased in abundance and 28 of which were decreased in abundance. ORM1 was markedly increased, and albumin was decreased in abundance. Cytoplasmic actin 1 and vitamin K-dependent protein were increased. Albumin, DCP2, hemoglobin subunit alpha, peroxiredoxin, and coagulation factor XIII B chain were decreased in abundance in NTM cases as compared to LTBI. Thirty-three proteins were increased and thirty-one proteins were decreased in abundance in active TB as compared to LTBI. Twenty-one proteins were increased in abundance and 22 proteins were decreased in abundance in active TB as compared to NTM. A cluster of proteins generally most abundant in active TB cases contained many acute phase reactants. Additional proteins are protein S100-A9 (S100-A9), sialic acid-binding Ig-like lectin 16 (SIGLEC16), protein S100-A8 (S100-A8), complement factor-1 (CF1), lactotransferrin (LTF) and vitamin K-dependent protein S (PROS1). Many of these proteins influence the chemotaxis and activation of leukocytes, often in conjunction with platelet degranulation.

### Proteins with significant differences in both stimulated and unstimulated plasma samples

**Fig. 3:**
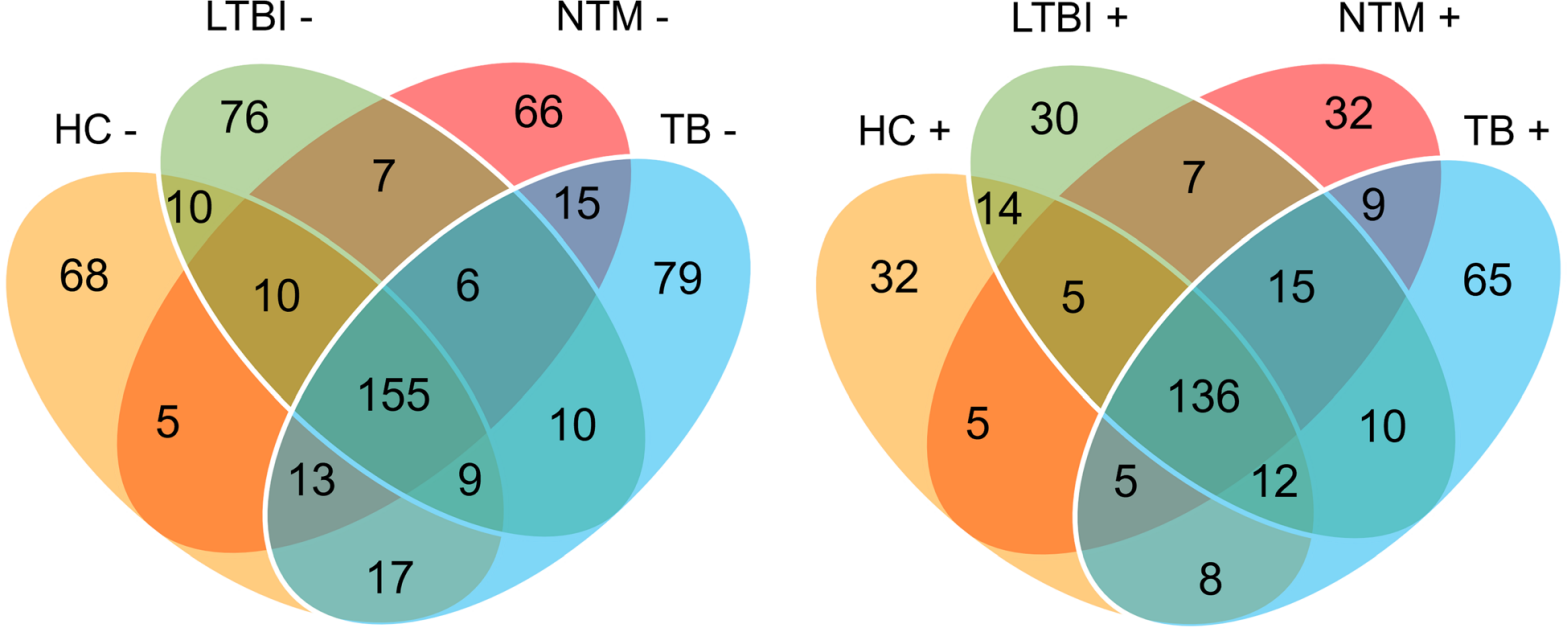
Protein identification overlaps among three disease and HC groups plotted in the Venn diagram. The (−) sign denotes no antigen stimulation and the (+) sign denotes antigen stimulation

**Fig. 4:**
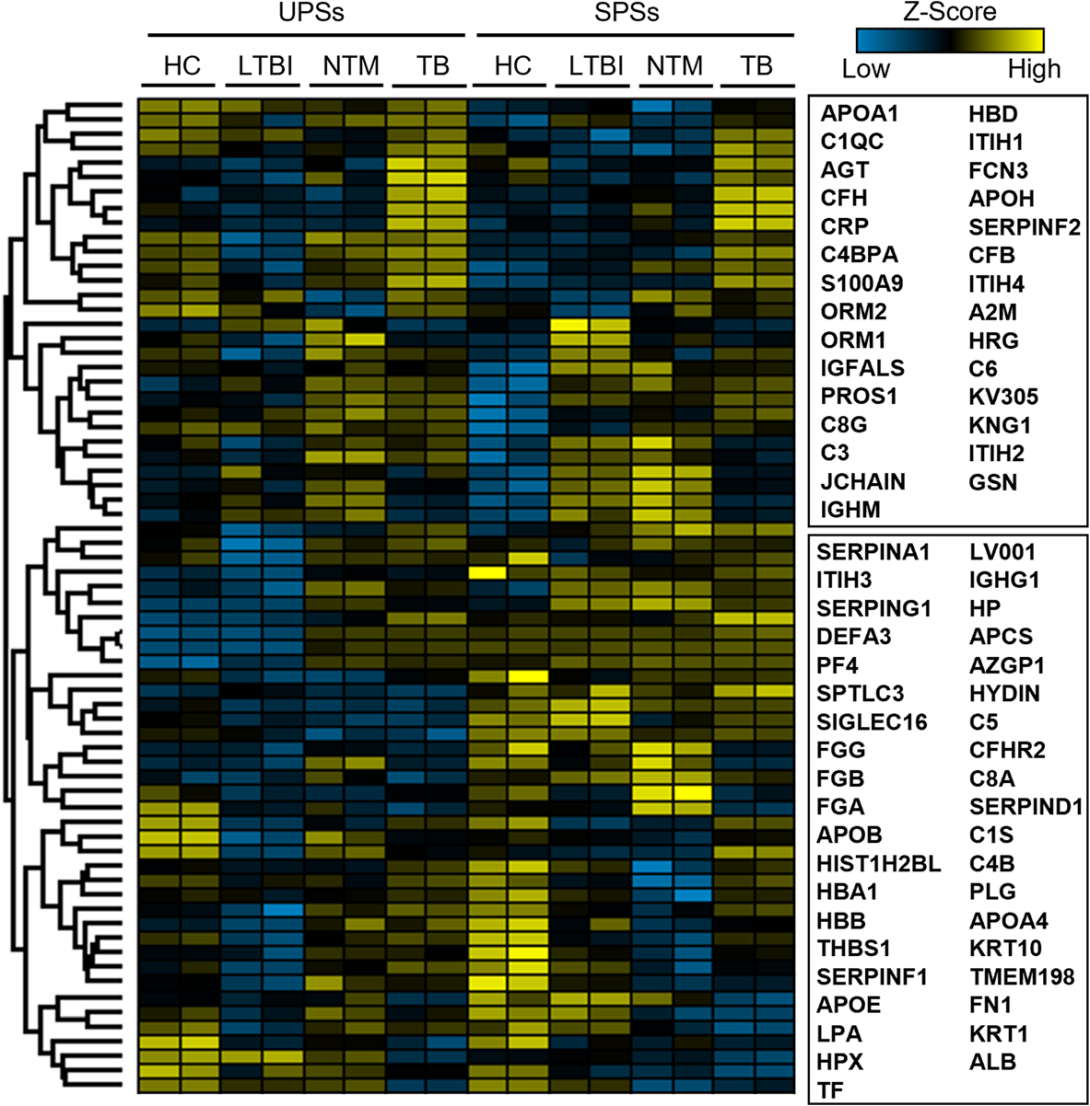
Heatmap of plasma protein subset with quantitative changes among the four groups. For the protein level analysis of 2 h and 6 h timepoints after gefitinib treatment only duplicate samples were available, limiting the possibility to perform statistical analysis of altered protein levels. Still, a heatmap visualization of the protein level quantification at all timepoints indicates a gradual increase/decrease of protein levels with clearly visible patterns already 2 h after EGFRTKI treatment significantly. The comparison of the signal levels for the 152 analytes measured by multiple-sample test (ANOVA) revealed 68 proteins significantly different among the eight groups with a Z-Score *>* 1.5, *p <* 0.05 with Benjamini-Horchberg false-discovery rate (FDR; *q*) of 0.05 correction. All 68 ANOVA significant proteins (Benjamini-Hochberg FDR 0.01 correction) were plotted here. The genes names of the proteins were displayed on the y-axis. Blue color indicates down regulation, black color indicates no change and yellow color indicates up regulation

**Fig. 5:**
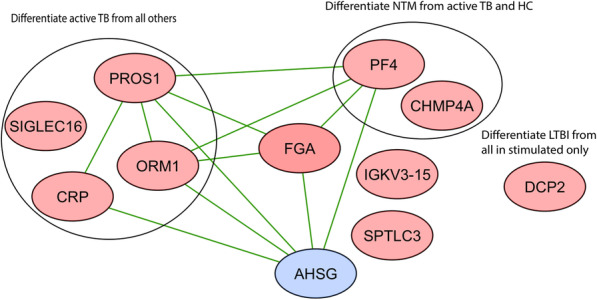
The black-lined circles on the left and right highlight proteins specifically abundance-changed in the TB group and those for the NTM vs. TB comparison, respectively. Those that are not encircled, differentiated LTBI from the other groups for SPS samples

The protein coverage of the four groups (HC, NTM, TB, and LTBI) with and without antigen stimulation is presented in Venn diagram-1 (Fig. 3). A total groups of 287, 283, 277, and 304 proteins were identified in HC, LTBI, NTM, and active TB in UPSs, of which 155 proteins were shared by all four groups. In SPSs, 210, 229, 214, and 260 proteins were identified in HC, LTBI, NTM, and active TB groups, of which 136 proteins were shared by all four groups**.** To identify the proteins that showed significant differences among the eight groups (HC, LTBI, NTM, and TB; with and without stimulation) a global ANOVA analysis was performed (Supplemental Table- S3). The analysis led to 68 significant proteins of which many were associated with coagulation (FGB, FGA, FGG, SPTLC3), the acute phase response (HP, CRP, ORM1, APCA), apolipoproteins (APOA1, APOA4, APOB, APOE and APOH), complement components (C1QC, C1S, C3, C4B, C5,C8A, C8G, CFB, CFH CFHR) and hemolysis (hemoglobin subunits) (Fig. 4). Further independent-sample *t*-tests were used to identify proteins that differentiate the four groups (HC, NTM, TB, and LTBI) in both UPSs and SPSs. DCP2 was increased in abundance in the LTBI groups as compared to the other three groups (TB, NTM, and HC) in USP and SPS datasets and not regulated in HC upon stimulation with the ESAT-6/CFP-10 cocktail; making the enzyme a potential biomarker candidate for the diagnosis of LTBI. CRP, ORM1, SIGLEC-16, and PROS1, all increased in abundance in both SPS and UPS (active TB versus in LTBI), are potential biomarkers for the diagnosis of active TB. Charged multivesicular body protein 4a (CHMP4A) and platelet factor (PF)-4 are up-regulated in NTM as compared to HC and down-regulated in NTM as compared to active TB. We hypothesize that a pattern of similar quantitiative protein changes in NTM and LTBI datasets reflects co-existance of LTBI with NTM in the community (Supplemental Table-S2). Platelet factor (PF)-4, Fibrinogen alpha chain (FGA), alpha-2-HS-glycoprotein, alpha-2-HS-glycoprotein chain A, alpha-2-HS- (AHSG), CRP, ORM1, and PROS1 have physical and/or functional interactions and constitute a network through STRING (Search Tool for the Retrieval of Interacting Genes/Proteins) database analysis (Fig. 5). Further independent-sample *t*-tests were used to identify proteins that were up or down-regulated upon stimulation with the ESAT-6/CFP-10 antigen cocktail. Twenty-nine proteins were up-regulated and 30 proteins were down-regulated in SPSs compared to USPSs in HC; 29 proteins were up-regulated and seven proteins were down-regulated in SPSs compared to UPSs in LTBI; two proteins were up-regulated and two proteins were down-regulated in SPSs compared to UPSs in NTM, and 6 proteins were up-regulated and 2 proteins were down-regulated in SPSs compared to UPSs in active TB (Supplemental Table-S4**).** The data are consistent with the notion that antigen stimulation had occurred in symptomatic patients (TB, NTM) as part of the immuno-physiological response, thus diminishing the extent of immune response-associated protein changes in plasma compared to SPSs. This is not the case for LTBI and, particularly, HC cohorts.

### Bioinformatic analysis

**Fig. 6:**
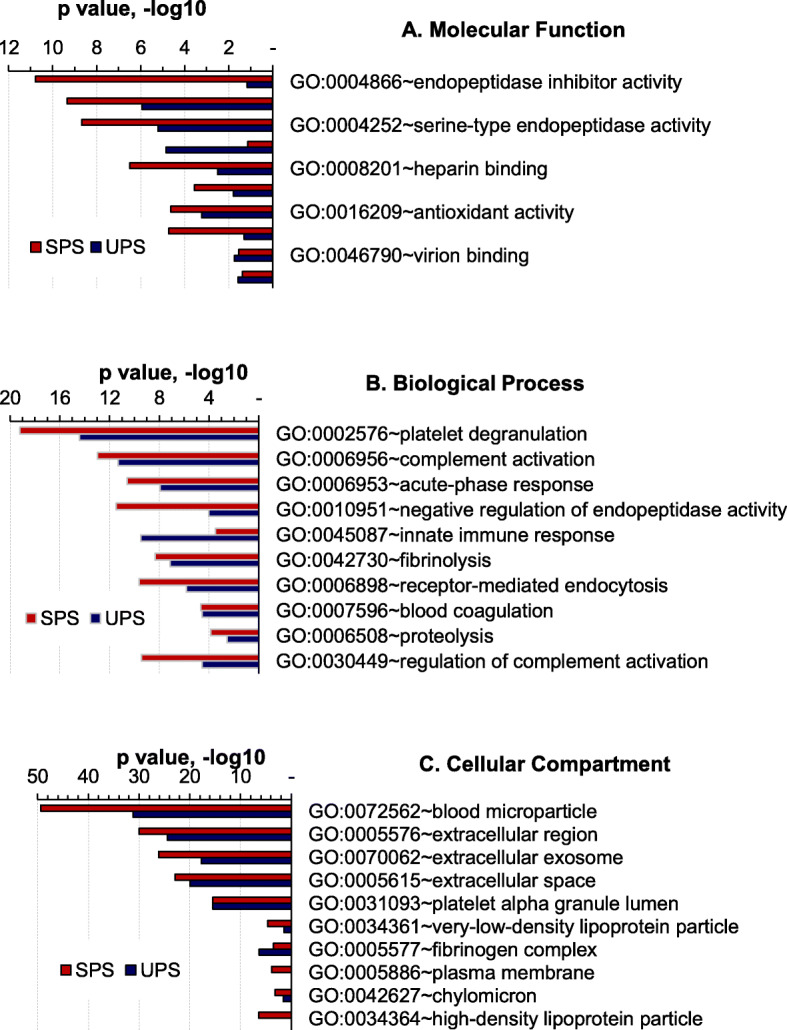
Functional analysis of protein categories using a bioinformatics resource. Differentially abundant proteins derived from the comparison of all three disease categories (TB, LTBI, NTM) compared to HC subjects were combined, separately for the UPS and SPS database, and subjected to Gene Ontology enrichment analysis for (**a**) molecular function, (**b**) biological process, and (**c**) cellular component, (http://geneontology.org/docs/go-enrichment-analysis/). Top ten most enriched GO terms are listed with their enrichment *P*-values

We used GO term ontology to assess the enrichment of biological entities (molecular functions, biological processes, cellular localization) based on the differentially abundant proteins (*P* < 0.05), data from all disease group comparisons with HCs were considered. With respect to cellular compartment analysis, the GO terms blood microparticle and extracellular region/exosome/space were highly enriched for UPS and SPS datasets, more so in SPS datasets. Microparticles (MPs) are considered to reflect cellular stimulation, activation, degeneration, and apoptosis [36], consistent with the notion that immune cells are directly stimulated with the TB antigens in SPSs but not in UPSs. Most blood plasma proteins are secreted proteins, explaining the enrichment of extracellular entities. With respect to molecular function analysis, the GO terms endopeptidase inhibitor activity and serine-type endopeptidase activity were highly enriched in SPSs but only moderately enriched in UPSs. Many acute-phase proteins including serpins are endopeptidase inhibitors. This is consistent with increased stress responses elicited by immune cell stimulation and the release of endopeptidases in SPSs upon activation by TB antigens. Antigen binding functions were more enriched in SPSs than in UPSs, consistent with the expected binding of ESAT-6 and CFP10 to MHC class II molecules for presentation to CD4 T-cells. It which results in IFN-γ production of the stimulated T-cells [37, 38]. In contrast, the term immunoglobulin receptor binding was much more enriched for USP datasets, supporting the notion of specific T-cell stimulation by the two TB antigens. With respect to biological processes, GO terms more enriched in SPSs had a regulatory context (negative regulation of endopeptidase activity and regulation of complement activation). This is in agreement with the need to regulate proinflammatory activities upon antigen stimulation. Indeed, proteins induced in SPSs had molecular function and biological process enrichments for the complement system, the acute phase response and endopeptidase activities (Fig. 6). Platelet degranulation was the most enriched GO term for SPS datasets, consistent with the role of platelets in immune cell stimulation.

## Discussion

The currently used diagnostic tests for active TB (such as microscopy and culture-based) are not sensitive enough and relatively slow for point of care applications [39, 40]. The currently used diagnostic test for LTBI, an IGRA, has generally poor specificity. Simultaneous measurement of multiple cytokines from individual T-cells is confounded by response variability due to potential co-stimulation of T-cell responses derived from other pathologies. Transcriptional signatures are difficult to incorporate into tests in the diagnostic setting [13]. Proteomics can improve our understanding of pathological processes involving interacting components of the immune system and signaling pathways [41]. In this study 189 proteins of which 34 proteins were differentially abundant among active TB*,* NTM, LTBI, and HC cohorts in UPSs, were quantified by label-free approach. In addition, 190 proteins of which 64 proteins were differentially abundant among active TB*,* NTM, LTBI, and HC cohorts were quantified in SPSs. Bioinformatics analysis showed that most of the differentially expressed proteins are involved in cellular processes such as responses to stimuli, metabolic processes, and biological regulations. Our findings are in agreement with a study conducted by Dan-Danet al [17] that showed that most abundance-changed proteins in TB patients (compared to a HC group) are involved in metabolic processes, responses to stimuli, and the immune system.

DCP2 is a decapping metalloenzyme that catalyzes the cleavage of the cap structure on mRNAs, specific to N^7^-methylated guanosine containing RNA, and thus influences mRNA turnover [39, 40]. The 5′ end of eukaryotic mRNA is capped and methylated to protect mRNA from degradation and enhance protein synthesis [42, 43]. The cap can be removed from mRNA resulting in GpppRNA. which is at risk of degrading mRNA former to splicing, export, and translation. The study conducted by Anna et al. [44] showed TbDcp2 is capable of releasing m7Gpp from m7GpppRNA in a magnesium-dependent manner. Uncapped RNA effectively inhibited Dcp2 activity. In this study, m7GpppN-mRNA hydrolase was increased in abundance in LTBI as compared to HC. M7GpppN-mRNA hydrolase was highly decreased in abundance in stimulated active TB as compared LTBI, which may indicate that stimulation with TB antigen up-regulate this protein in LTBI. More surprising, the protein was not changed upon stimulation with ESAT-6/CPF-10 on HC and NTM; this suggests a selective protein abundance change for infection with MTBC. To our knowledge, DCP2 was not reported as involved in the host response to TB elsewhere. However, it was reported that host gene-environment interactions play a crucial role in determining the outcome of TB. Such interactions are considered important to evolve strategies to prevent mycobacterial infection [45]. Functional and biomarker validation studies are needed to elucidate the role of DCP2 in TB and LTBI.

A cluster of five proteins more abundant in TB compared to all other groups has a strong functional association with the acute phase response. CRP and ORM1 are the most common acute-phase proteins relevant to infections as they bind pathogens and facilitate complement activation [46]. CRP promotes phagocytosis and complement fixation through its calcium-dependent binding to phosphorylcholine, and its levels rise in active TB infection through IL-6 mediated fusion [47]. In this study, CRP was increased in abundance in both SPS and UPSs in patients with active TB as compared to HC, LTBI, and NTM. Our data are in agreement with other results [16, 48, 49], especially in the context of CRP abundance. ORM1 is an acute-phase protein promoting disease progression by suppressing cell-mediated immunity and thereby enhancing the growth of bacilli [50–52]. The main sources of ORM1 are alveolar macrophages and type II pneumocytes at the early stage of pulmonary TB. At advanced stages, foamy macrophages locate in tuberculous areas [50, 53]. Pulmonary inflammation increases the ORM1 concentration in the serum and inflamed areas of the lungs [50]. Multiple studies on primary cultures of rat and human hepatocytes indicated that low levels of albumin and high levels of ORM1 are attributed to the regulation of monokines, particularly IL-6 [54–56]. In our previous study [21], IL-6 did not show quantitative differences comparing active TB with LTBI as well as HC. We conclude that CRP and ORM1 are important mediators of the stress response to immune activation and monokine-associated inflammation in active TB cases.

Increased abundances of proteins that are involved in platelet degradation (as determined by our study) may indicate the role of platelets in antimycobacterial immunity. Our data agree with the other studies [57] where platelets were associated with anti-mycobacterial immunity in parallel to macrophages and IFN-γ producing T-lymphocytes [58]. Platelets are increasingly recognized to have diverse functions in inflammation and host defense [59]. Platelets also interact extensively with leukocytes and change phenotype and functions of the latter [60–62] through ligation of platelet P-selectin with monocyte P-selectin glycoprotein ligand-1 (PSGL-1) [63]. Studies showed that platelets are important players in the formation and function of granuloma and macrophage transformation in TB [64]. Platelets also play a role in facilitating migration of monocytes into tissues [65], through up-regulation of CCR5 during TB infection via preferentially bind to CD16 + monocytes [66]. Increased abundance of PROS in both SPSs and USPs in active TB as compared to LTBI (our data) suggests that patients with TB are in a systemic hypercoagulable state [67]. Our data argue for complement system activation, especially for the TB cohort, following stimulation by EST-6/CFP10.We hypothesize that the stimulation with TB antigens activates the complement cascade and platelets which, in turn, activate leukocytes. Leukocytes produce calgranulins (S100-A8 and S100-A9) which contribute to the release of cytokines [68]. Cytokines were not detected due to concentration ranges below the limit of detection for LC-MS/MS plasma proteomics.

Chronic pulmonary disease is the most common clinical manifestation of NTM. Physical findings and symptoms-based clinical examination of NTM pulmonary disease are variable and nonspecific. AFB microscopic examination is also not specific, as evidenced by 16.9% of the *Mycobacterium* isolates in respiratory samples that were NTM positive for AFB [69]. Presumptive diagnosis based on clinical and radiographic features is not adequate for the initiation of therapy. Though the excellence of IGRA ELISA to differentiate NTM and TB infection in non-endemic countries was reported [70], 34–49% IGRA ELISA positive cases were reported in NTM in endemic countries [71]; this supports our hypothesis that the similarity of protein changes observed for both LTBI and NTM cases are explained by frequent coexistence of LTBI and NTM infection in our study community. The proteins of NTM that were identified in this study and previously described by Jusus et al. [72] appear to reflect cross-reactivity observed between MTBC and NTM exposition. Two proteins such as the charged multivesicular body protein 4a (CHMP4A) and the platelet factor (PF)-4, were up-regulated in NTM as compared to HC and down-regulated in NTM as compared to active TB in this study. CHMP4A belongs to the chromatin-modifying protein/charged multivesicular body protein (CHMP) and components of ESCRT-III (endosomal sorting complex required for transport III). The latter is a complex involved in the degradation of surface receptor proteins and the formation of endocytic multivesicular bodies (MVBs) [73]. The down-regulation of CHMP4A in NTM as compared to active TB supports the notion that NTM infection is prevalent in immunocompromised individuals [74].

## Conclusion

Plasma proteomes are different comparing LTBI, TB and healthy groups and change further upon ESAT-6/CFP-10 antigen cocktail stimulation. Further studies using larger sample sizes are warranted to validate the robustness and potential clinical value of our identified TB biomarkers. Upon validation, a subset of those may serve to develop tests for rapid clinical diagnosis of TB infected individuals.

## Supplementary information

**Additional file 1: Supplemental dataset S1.** The summary file contains summary information for all the raw files processed with a single MaxQuant run. The summary information consists of some MaxQuant parameters, information of the raw file contents, and statistics on the peak detection. Based on this file a quick overview can be gathered on the quality of the data in the raw file. The clustering and correlation analyses were performed in Perseus environment (version 1.5.0.15) using embedded functions. The LFQ intensities generated by MaxQuant were log (base 2) transformed, and then imputed with missing values by default settings in Perseus. LC-MS/MS data were deposited to the Proteome Xchange Consortium via the PRIDE partner repository with the dataset identifier PXD012412. The letters A and B denote biological replicates (later merged together), and rep1 and rep2 denote technical replicate. Number 1–4 are unstimulated plasma samples and number 5–8 are stimulated plasma samples.

**Additional file 2: Supplemental dataset S2.** Differentially abundance values of proteins comparing four groups (HC, LTBI, NTM and active TB) to each other’s measured from analyzed in two technical replicates computing the abundances with the MaxLFQ software tool. The analytes were measured from 40 enrolled participants (10 each for the cohorts HC, LTBI, active TB and NTM) from unstimulated and 40 enrolled participants (10 each for the cohorts HC, LTBI, active TB and NTM) from stimulated. Thirty-five proteins were differentially expressed in LTBI comparing to HC, 43 proteins were differentially expressed in NTM comparing to HC and 49 proteins were differentially expressed in active TB comparing to HC, 34 proteins were differentially expressed in NTM as compared to LTBI, 41 proteins were differentially expressed in active TB as compared to LTBI, and 39 proteins were differentially expressed in active TB as compared to NTM in unstimulated plasma samples. In the meanwhile, 50 proteins were differential expressed in LTBI comparing to HC, 41 proteins were differential expressed in NTM comparing to HC, 61 were differentially expressed in active TB comparing to HC, 13 proteins were differentially expressed in NTM as compared to LTBI, 64 proteins were differentially expressed in active TB as compared to LTBI, and 43 proteins were differentially expressed in active TB as compared to NTM. Independent-sample t testing was performed and *P*-values < 0.05 were considered statistically significant. Student’s t-test difference of + 1 or − 1 was used as cutoff value. False discovery rate was calculated and q-values ≤0.05 were also considered statistically significant. Columns in the Excel files designate technical replicates (donated by rep), comparison groups, peptides, T-test difference, t-test values, q-values, protein ID, protein names and gene names. Significantly up-regulated proteins were highlighted by red color, significantly down-regulated proteins were highlighted by blue color, non-significantly up and down regulated proteins were highlighted by yellow color and not regulated proteins were highlighted by black color.

**Additional file 3: Supplemental database 3.** Global ANOVA analysis to identify the proteins that showed significant differences among the eight groups (HC, LTBI, NTM, and TB) in plasma samples with and without stimulation. To perform the quantization analysis, the LFQ values of each protein derived from MaxQuant were first log2 transformed and filtered to eliminate proteins with the most missing values. Missing values for other proteins were imputed and then multiple-samples test (ANOVA) was performed with Benjamini-Hochberg correction (FDR = 0.05), which led to 68 proteins that showed significant difference among the eight groups. *P*-values < 0.05 were considered statistically significant. Columns in the Excel files designate technical replicates (donated by rep), comparison groups, peptides, MS/MS count, ANOVA P-value, ANOVA q-value, T-test difference, t-test values, q-values, protein ID, protein names and gene names.

**Additional file 4: Supplemental dataset S4.** Independent-sample *t*-tests to identify proteins that were up-regulated or down-regulated upon stimulation with the ESAT-6/CFP-10 antigen cocktail. Fifty-nine proteins were significantly regulated upon stimulation with the ESAT-6/CFP-10 antigen cocktail in HC, 36 proteins were significantly regulated upon stimulation with the ESAT-6/CFP-10 antigen cocktail in LTBI, and four proteins were differently regulated upon stimulation with the ESAT-6/CFP-10 antigen cocktail in NTM, eight proteins were significantly regulated upon stimulation with the ESAT-6/CFP-10 antigen cocktail in active TB. Columns in the Excel files designate technical replicates (donated by rep), t-test significant, comparison groups, *P*-value, q value, t-test difference, protein ID, protein names and gene names. *P*-values < 0.05 were considered statistically significant.

## Abbreviations

AHSG: Alpha-2-HS-glycoprotein;Alpha-2-HS-glycoprotein chain A;Alpha-2-HS-
ANOVA: Analysis of variance
APCS: Serum amyloid P-component;Serum amyloid P-component (1–203)
BCG: Bacilli Calmette Guerin
CHMP4A: Charged multivesicular body protein 4a
CCR: CC Chemokine receptor 5
CFH: Complement factor H
CFP-10: Culture filter protein-10
CO_2_: Carbon dioxide
CRP: C-reactive protein
DCP2: M7GpppN-mRNA hydrolase
DNA: Deoxyribonucleic acid
ETAT-6: Early secretary antigenic target-6.
FASP: Filter aided sample preparation
FDR: False discovery rate
FGA: Fibrinogen alpha chain
GO: Gene Ontology
HC: Healthy control
HCD: High-energy collisional dissociation
HIV: Human immunodeficiency
IFN-γ: Interferon Gamma
IgA: Immunoglobulin alpha
IgG: Immunoglobulin gamma
IgM: mmunoglobulin
IGRA: Interferon Gamma Release Assays
IL: Interleukin
kDa: Kilo Dalton
LC-MS/MS: Liquid chromatography mass spectrometry
LFQ: Label-free quantitative
LGALS3B: Galectin-3-binding protein
LSD: Least significant difference
LTBI: Latent TB infection
MPs: Microparticles
mRNAs: Messenger ribonucleic acids
Mtb: *Mycobacterium tuberculosis*
NTM: Non-tuberculosis mycobacteria
ORM1: α-1-acid glycoprotein 1
PF: Platelet factor
PHA: Phytohemagglutinin antigen
PPD: Purified protein derivation
PROS1: Vitamin K-dependent protein S
PSGL-1: P-selectin glycoprotein ligand-1
QFT-IT: QuantiFRON-TB Gold In-Tube test
RPMI: Roswell Park Memorial Institute
S100-A8: Protein S100-A8;Protein S100-A8, N-terminally processed
SD: Standard deviation
SDS-FAGE: Sodiumdedecyle sulfate-Polyacrylamide gel electrophoresis
SERPINA1: Alpha-1-antitrypsin;Short peptide from AAT
SIGLEC16: Sialic acid-binding Ig-like lectin 16
SPSs: Stimulated plasma samples
SPTLC: Serine palmitoyl transferase
STRING: Search tool for the retrieval of interacting genes/proteins
TB: Tuberculosis
TPM4: Tropomyosin alpha-4 chain
UPSs: Unstimulated plasma samples
χ^2^: Chi-square
